# Supplementary material for: Molecular and epistatic interactions between pioneer transcription factors shape nucleosome dynamics and cell differentiation
Source: Nat Commun. 2025 Dec 10;17:698. doi: 10.1038/s41467-025-67308-0 (PMC12820075; doi:10.1038/s41467-025-67308-0)
Supplement: Supplementary file 1 — Supplementary Information [file 41467_2025_67308_MOESM1_ESM.pdf]

R.X. Coux et al.

Supplementary Figures

Contents:

**Page 2**	**Figure S1.** Conversion of ES cells into PrE cells by GATA6 induction.
**Page 3**	**Figure S2.** Statistical analyses of GATA6 binding regions.
**Page 4**	**Figure S3.** Analysis of SOX17, a target and partner of GATA6.
**Page 5**	**Figure S4.** Impact of SOX17 in GATA6 binding and chromatin dynamics.
**Page 6**	**Figure S5.** Comparative analysis of GATA6 and pluripotency TF binding regions.
**Page 7**	**Figure S6.** OCT4 responsive genes during GATA6-mediated PrE differentiation.
**Page 8**	**Figure S7.** Stratification and gene expression correlations of GATA6 binding regions.

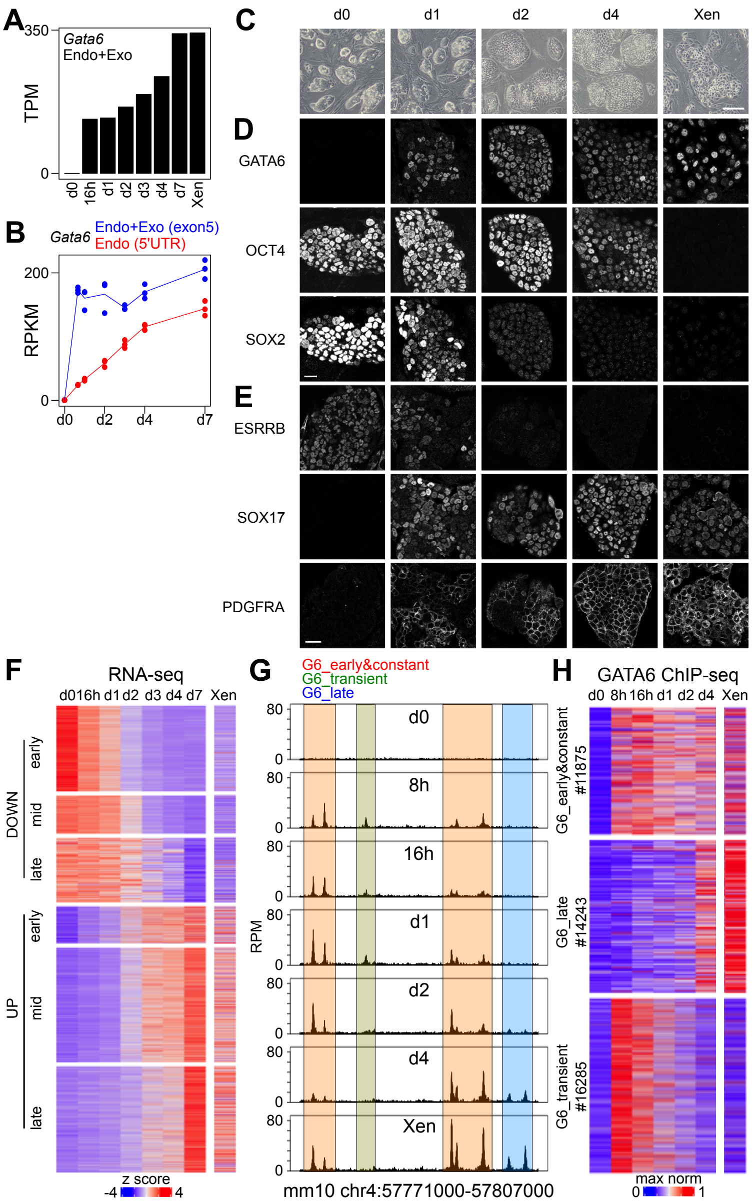

Figure S1. Conversion of ES cells into PrE cells by GATA6 induction. (A) Expression (Transcripts Per Million) of *Gata6* mRNA (endogenous plus exogenous) during GATA6 induction and in wild-type XEN cells. **(B)** Comparative quantification of the last *Gata6* exon 5, present in both endogenous and exogenous *Gata6* transcripts, and of its 5' UTR, present only in endogenous mRNAs, normalized in Reads Per Kb Per Million. **(C)** Illustrative photomicrograph of ES cells (d0) and as they convert into PrE cells following Dox treatment for 1, 2 and 4 days, as well as of XEN cells. The scale represents 200 µm. **(D)** Representative immunostaining of GATA6, OCT4 and SOX2 in the same samples as in (C). The scale represents 30 µm. **(E)** Representative immunostaining of ESRRB, SOX17 and PDGFRA in the same samples as in (C). The scale represents 30 µm. **(F)** Expression changes of the 6 groups of genes identified as early/mid/late up- or downregulated. **(G)** Illustrative locus showing different behaviors of GATA6 binding dynamics. **(H)** Relative GATA6 binding levels across the 3 clusters of GATA6 binding regions. For each region, the sample with maximal enrichment levels was set to 1.

Figure S2. Statistical analyses of GATA6 binding regions. (A) Several properties are considered and the regions divided in groups, from top to bottom: accessibility (small ATAC fragments), with regions divided as high, mid or low accessibility; the quality of the best motifs of GATA6, SOX17, OCT:SOX or ESRRB present in each region measured by their score, with the regions divided as presenting motifs with high, mid, low scores or none at all; histone modification status as evaluated by quantification and clustering of Encode data, grouping the regions according to the presence of marks typical of distinct regulatory states, shown in the right (F. facultative; C. constitutive). The boxplots show the median – bar; 25-75% percentiles – box; 1.5-folds the inter-quartile range – whiskers **(B)** Proportion of the different groups of variables shown in (A) throughout Early&Constant, Late and Transient GATA6 binding regions. Variables dependency was shown to be significant using Chi-square tests for all combinations of variables ($p < 2.2e-16$). **(C)** Log2 Odds ratio for the combination of categories shown within each plot, derived from the variables shown on the left. Fisher Exact tests revealed the statistical significance of all enrichments/depletions ($p<1.3e-6$). **(D)** The variables shown in (A) were tested as predictors for each region belonging to one of the 3 GATA6 binding groups (Early&Constant, Late, Transient). The importance of each feature is shown on the left and the predictive success on the right, reaching an Area Under the Curve of 0.81. See Methods for details.

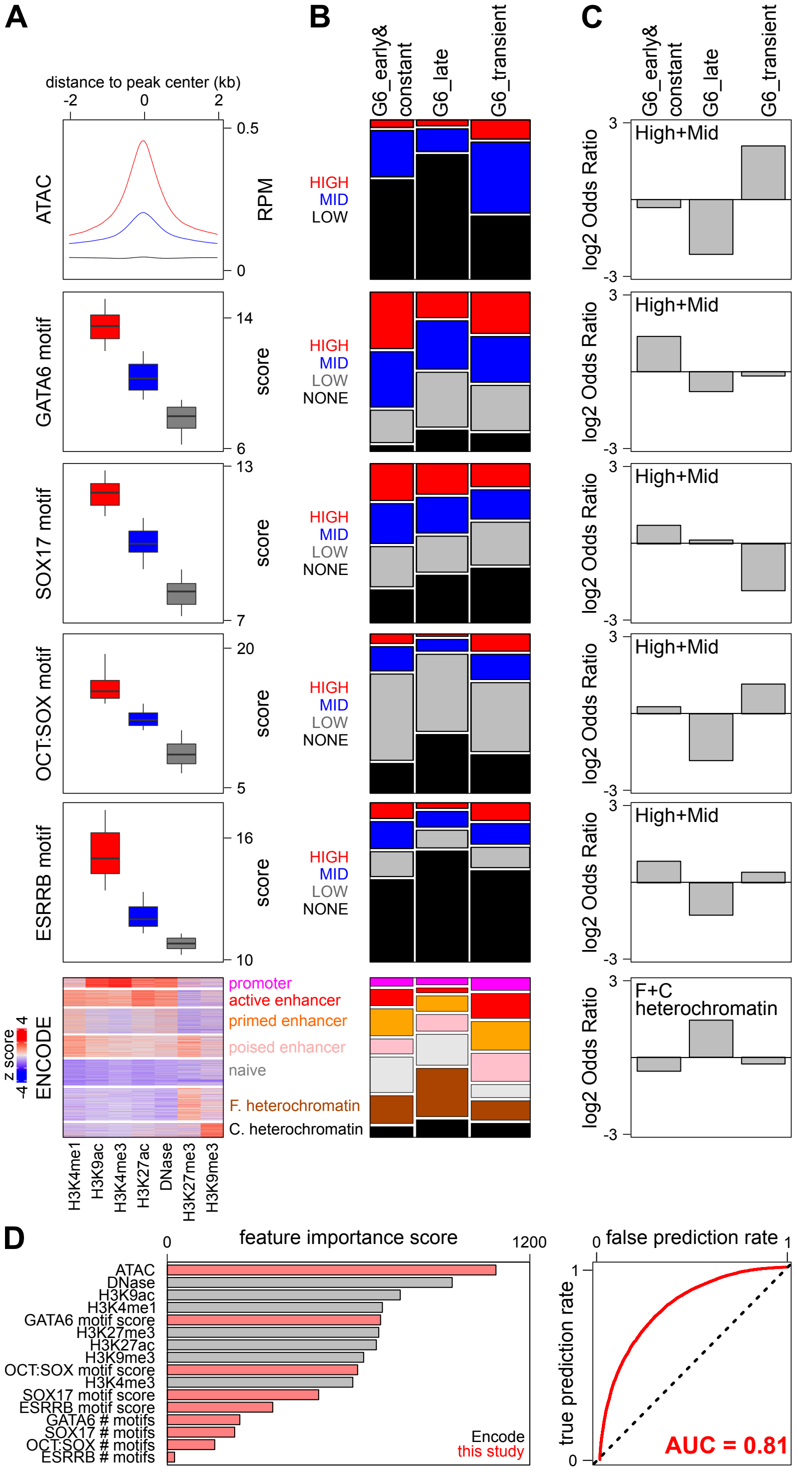

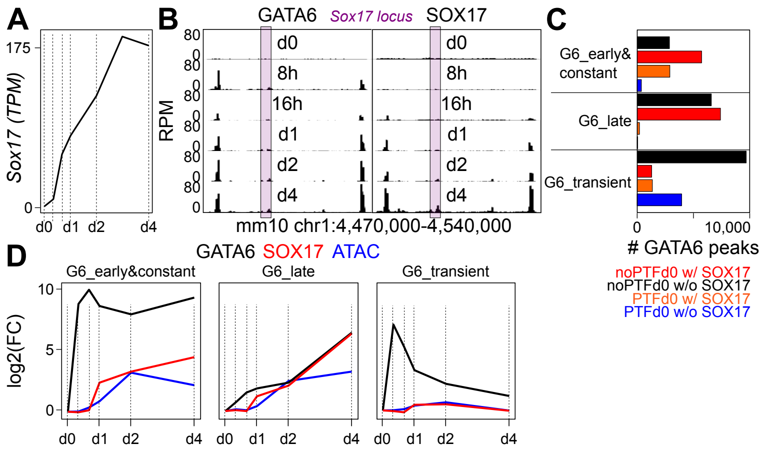

Figure S3. Analysis of SOX17, a target and partner of GATA6. (A) RNA-seq expression profile of Sox17 mRNA expressed in Transcripts Per Million (tpm) over GATA6 induction. **(B)** Illustrative co-binding of GATA6 and SOX17 around Sox17 (purple box). **(C)** Distribution of GATA6 peaks across different categories based on the dynamics of GATA6 binding (Early&Constant, Late, Transient) and the presence/absence of either pluripotency TFs before differentiation (PTFd0/noPTFd0) or presence (w/) or absence (w/o) of SOX17. **(D)** Average log2 Fold-Change of chromatin accessibility, GATA6 and SOX17 binding at every time-point of GATA6 induction versus undifferentiated cells (d0).

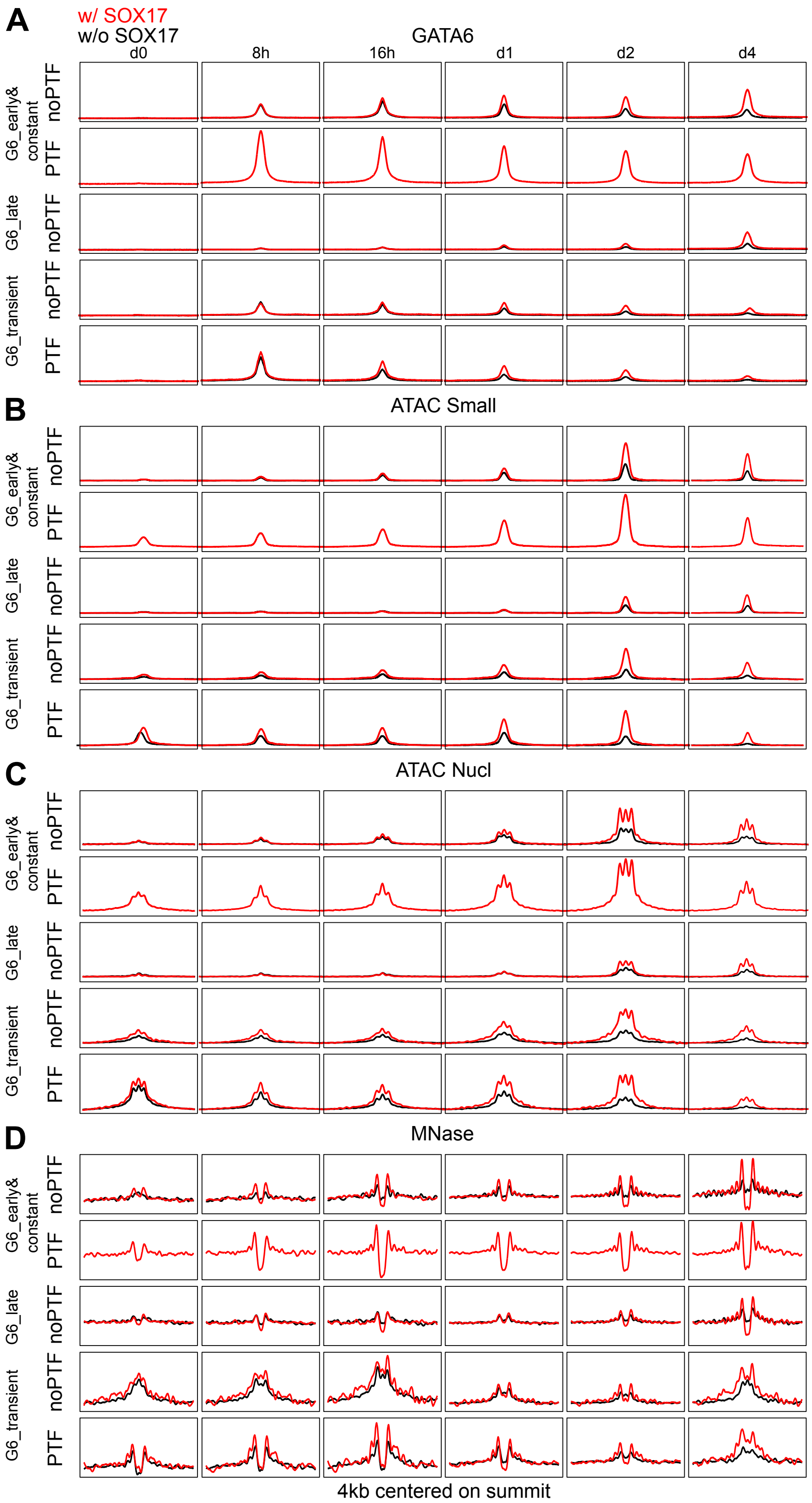

Figure S4. Impact of SOX17 on GATA6 binding and chromatin dynamics. The groups capturing most regions in Fig.S3C were used to compare GATA6 binding **(A)**, chromatin accessibility using small ATAC-seq fragments **(B)** and nucleosomes using nucleosome-sized ATAC-seq fragments **(C)** or MNase-seq **(D)**. The plots show the average reads per million across 4kb centered on GATA6 summits for the regions shown vertically that either bind SOX17 (red) or not (black).

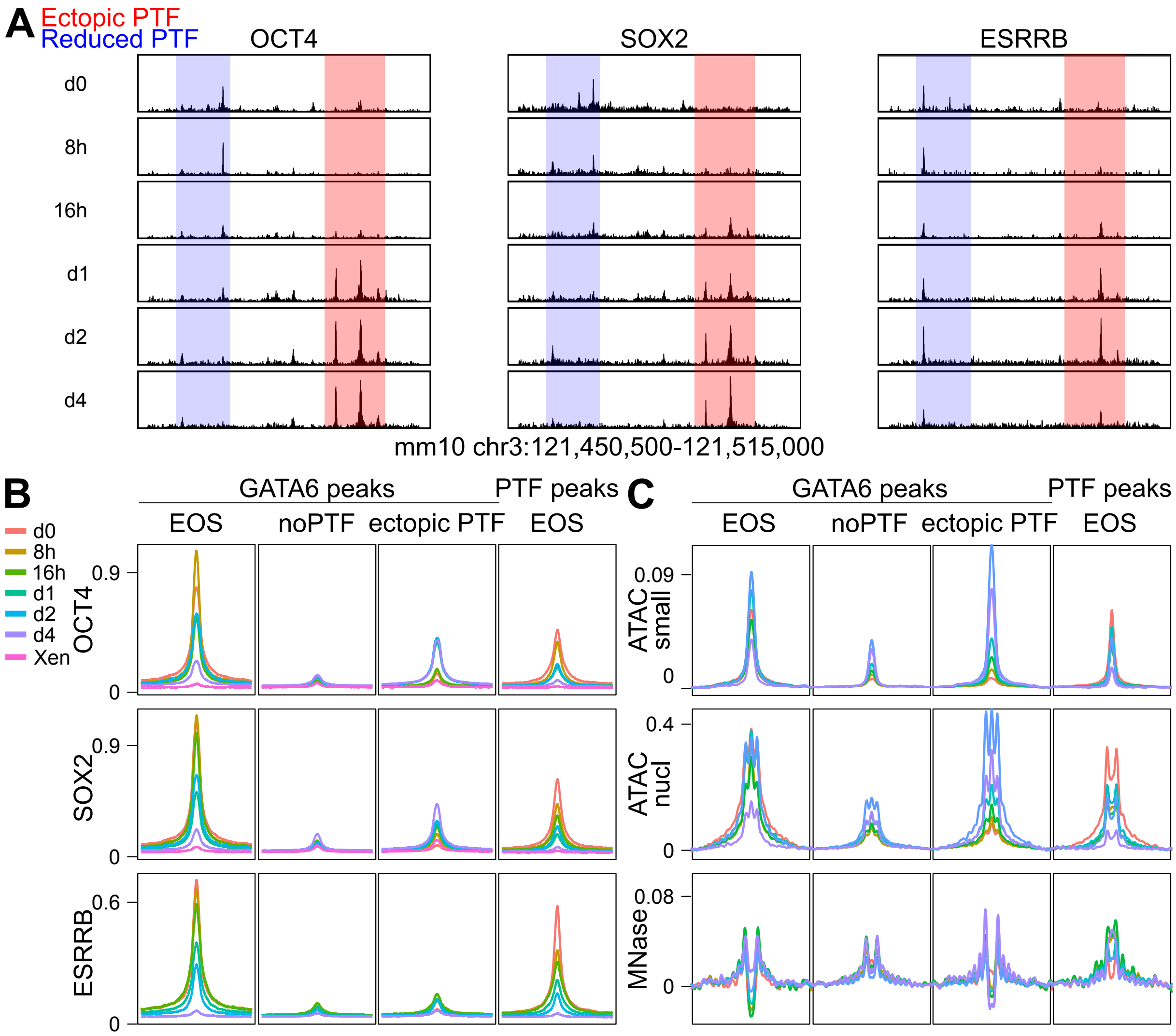

Figure S5. Comparative analysis of GATA6 and pluripotency TF binding regions. (A) Example of pluripotency TF binding during GATA6 induction showing regions losing or ectopically gaining their binding. **(B)** Average binding profiles of pluripotency TF at GATA6 and pluripotency TF (PTF) binding peaks throughout all analyzed time-points. Each set of regions was divided according to the binding of either all 3 analyzed pluripotency TFs in undifferentiated cells (EOS for ESRRB, OCT4 and SOX2), none of the three or when showing ectopic binding during GATA6 induction (ectopic PTF). The regions shown represent 4kb centered on the GATA6 summit for GATA6 peaks or on the summit of chromatin accessibility for PTF peaks. **(C)** Identical analyses for chromatin accessibility measured by small ATAC-seq framents or for nucleosomes measured by nucleosome-sized ATAC-seq or MNase-seq fragments.

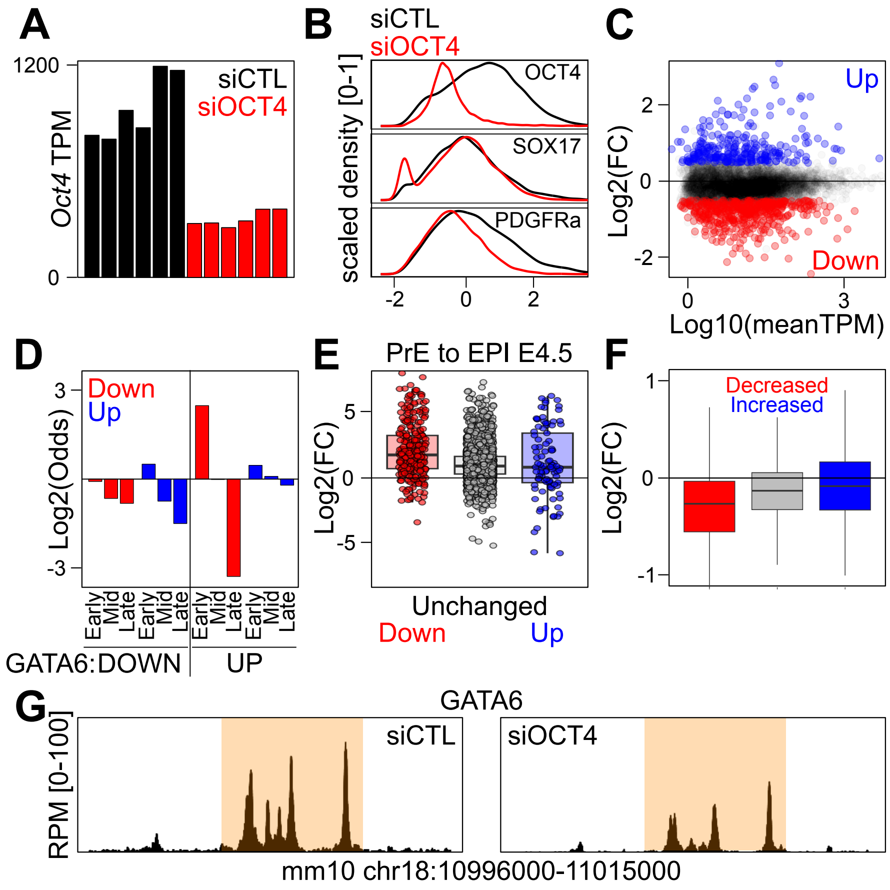

Figure S6. OCT4 responsive genes during GATA6-mediated PrE differentiation. (A) Expression of *Oct4* 48h after inducing GATA6 and nucleofecting cells with either Ctl siRNAs (black) or siRNAs targeting *Oct4* (red). Six independent experiments are shown. **(B)** Quantification of immunofluorescence experiments 4d after inducing GATA6 in the presence of either Ctl or *Oct4*-targetting siRNAs. **(C)** MA-plot depicting all GATA6-responsive genes, highlighting those upregulated (blue) or downregulated (red) upon *Oct4* knock-down. **(D)** Log2 Odds Ratio computed between genes down or upregulated upon *Oct4* knock-down and the 6 groups of GATA6-responsive genes (X-axis). **(E)** Gene expression changes during *in vivo* differentiation to PrE, for genes up- or downregulated upon *Oct4* knock-down. **(F)** Global expression changes of the nearest gene to each GATA6 peak classified by its response to *Oct4* knock-down. **(G)** GATA6 binding across the PrE-specific *Gata6* enhancer after Ctl or *Oct4* knock-down.

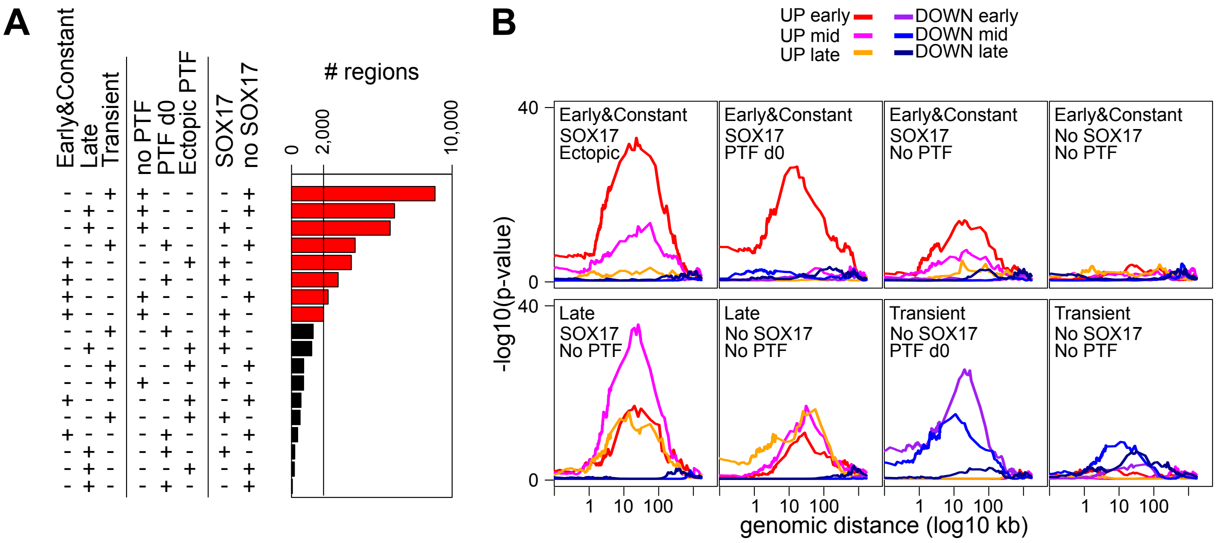

Figure S7. Stratification and gene expression correlations of GATA6 binding regions. (A) Repartition of all GATA6 binding regions across all previously studied variables (18 different combinations). The number of regions within each category is shown on the barplot. All combinations with more than 2000 regions, summing up to 85% of all GATA6 binding regions, were selected to build the scenarios shown in Fig.7. **(B)** Statistical association of the 8 major groups of regions shown in (A) with the 6 clusters of differentially expressed genes shown in Fig.1B, measured with Fisher Exact tests at increasing distances.
