# Supplementary material for: Molecular and epistatic interactions between pioneer transcription factors shape nucleosome dynamics and cell differentiation
Source: Nat Commun. 2025 Dec 10;17:698. doi: 10.1038/s41467-025-67308-0 (PMC12820075; doi:10.1038/s41467-025-67308-0)
Supplement: Supplementary file 2 — Reporting Summary [file 41467_2025_67308_MOESM2_ESM.pdf]

nature portfolio

Corresponding author(s): Cohen-Tannoudji and Navarro

Last updated by author(s): Nov 3, 2025

Reporting Summary

Nature Portfolio wishes to improve the reproducibility of the work that we publish. This form provides structure for consistency and transparency in reporting. For further information on Nature Portfolio policies, see our Editorial Policies and the Editorial Policy Checklist.

Statistics

For all statistical analyses, confirm that the following items are present in the figure legend, table legend, main text, or Methods section.

n/a	Confirmed	
☐	☒	The exact sample size (n) for each experimental group/condition, given as a discrete number and unit of measurement
☐	☒	A statement on whether measurements were taken from distinct samples or whether the same sample was measured repeatedly
☐	☒	The statistical test(s) used AND whether they are one- or two-sided<br>*Only common tests should be described solely by name; describe more complex techniques in the Methods section.*
☒	☐	A description of all covariates tested
☐	☒	A description of any assumptions or corrections, such as tests of normality and adjustment for multiple comparisons
☐	☒	A full description of the statistical parameters including central tendency (e.g. means) or other basic estimates (e.g. regression coefficient) AND variation (e.g. standard deviation) or associated estimates of uncertainty (e.g. confidence intervals)
☐	☒	For null hypothesis testing, the test statistic (e.g. F, t, r) with confidence intervals, effect sizes, degrees of freedom and P value noted<br>*Give P values as exact values whenever suitable.*
☒	☐	For Bayesian analysis, information on the choice of priors and Markov chain Monte Carlo settings
☒	☐	For hierarchical and complex designs, identification of the appropriate level for tests and full reporting of outcomes
☒	☐	Estimates of effect sizes (e.g. Cohen's d, Pearson's r), indicating how they were calculated

Our web collection on statistics for biologists contains articles on many of the points above.

Software and code

Policy information about availability of computer code

Data collection	all data was generated internally or gathered from previous publications and public repositories as indicated in the manuscript.
Data analysis	All analyses were performed with standard packages (R version 3.6.3, bamsignals package, ggplot2, DEseq2, ComplexHeamtaps, Bowtie2, MACS2, RSEM, STAR).

For manuscripts utilizing custom algorithms or software that are central to the research but not yet described in published literature, software must be made available to editors and reviewers. We strongly encourage code deposition in a community repository (e.g. GitHub). See the Nature Portfolio guidelines for submitting code & software for further information.

Data

Policy information about availability of data

All manuscripts must include a data availability statement. This statement should provide the following information, where applicable:

- Accession codes, unique identifiers, or web links for publicly available datasets
- A description of any restrictions on data availability
- For clinical datasets or third party data, please ensure that the statement adheres to our policy

all RNA-seq, ATAC-seq, MNase-seq and ChIP-seq raw data have been deposited into Gene Expression Omnibus under the public accession number GSE268719

Research involving human participants, their data, or biological material

Policy information about studies with human participants or human data. See also policy information about sex, gender (identity/presentation), and sexual orientation and race, ethnicity and racism.

Reporting on sex and gender	NA
Reporting on race, ethnicity, or other socially relevant groupings	NA
Population characteristics	NA
Recruitment	NA
Ethics oversight	NA

Note that full information on the approval of the study protocol must also be provided in the manuscript.

Field-specific reporting

Please select the one below that is the best fit for your research. If you are not sure, read the appropriate sections before making your selection.

☒ Life sciences ☐ Behavioural & social sciences ☐ Ecological, evolutionary & environmental sciences

For a reference copy of the document with all sections, see nature.com/documents/nr-reporting-summary-flat.pdf

Life sciences study design

All studies must disclose on these points even when the disclosure is negative.

Sample size	The number of replicates used in the study are standard in the field.
Data exclusions	no dataset was arbitrarily excluded
Replication	all replicates are described in the methods
Randomization	randomization was not deemed necessary due to the implementation of orthogonal methods (ChIP, ATAC, MNase and RNA-seq), strategically chosen to consolidate key results and control for potential confounding variables
Blinding	All data was generated and analysed without prior knowledge of the biological nature of the groups

Reporting for specific materials, systems and methods

We require information from authors about some types of materials, experimental systems and methods used in many studies. Here, indicate whether each material, system or method listed is relevant to your study. If you are not sure if a list item applies to your research, read the appropriate section before selecting a response.

Materials & experimental systems

n/a	Involved in the study	
☐	☒	Antibodies
☐	☒	Eukaryotic cell lines
☒	☐	Palaeontology and archaeology
☒	☐	Animals and other organisms
☒	☐	Clinical data
☒	☐	Dual use research of concern
☒	☐	Plants

Methods

n/a	Involved in the study	
☐	☒	ChIP-seq
☒	☐	Flow cytometry
☒	☐	MRI-based neuroimaging

Antibodies

Antibodies used	anti-GATA6 (Cell Signaling technology #5851); anti-OCT4 (abcam ab19857); anti-SOX2 (Active Motif 39823); anti-ESRRB (Perseus Proteomics, H6-705-00); anti-SOX17 (R&D Systems AF1924)

Validation	all antibodies are commercially available, highly validated by the manufacturers and routinely used in the field. Our own immunofluorescence data validates their specificity

Eukaryotic cell lines

Policy information about cell lines and Sex and Gender in Research

Cell line source(s)	KH2 cells are an ES cell line widely used in the field to generate inducible single-copy trangenes (https://doi.org/10.1002/gene.20180). E14Tg2A is a standard cell line in ES research, originally obtained from Ian Chambers and Phil Avner. X10 XEN cells (10.1242/dev.050864) are an extraembryonic endoderm stem cell line commonly used and obtained from Jerome Artus.
Authentication	All cell lines were tested by genotyping
Mycoplasma contamination	All cell lines were tested negative by PCR
Commonly misidentified lines (See ICLAC register)	NA

Plants

Seed stocks	*Report on the source of all seed stocks or other plant material used. If applicable, state the seed stock centre and catalogue number. If plant specimens were collected from the field, describe the collection location, date and sampling procedures.*
Novel plant genotypes	*Describe the methods by which all novel plant genotypes were produced. This includes those generated by transgenic approaches, gene editing, chemical/radiation-based mutagenesis and hybridization. For transgenic lines, describe the transformation method, the number of independent lines analyzed and the generation upon which experiments were performed. For gene-edited lines, describe the editor used, the endogenous sequence targeted for editing, the targeting guide RNA sequence (if applicable) and how the editor was applied.*
Authentication	*Describe any authentication procedures for each seed stock used or novel genotype generated. Describe any experiments used to assess the effect of a mutation and, where applicable, how potential secondary effects (e.g. second site T-DNA insertions, mosiacism, off-target gene editing) were examined.*

ChIP-seq

Data deposition

☒ Confirm that both raw and final processed data have been deposited in a public database such as GEO.

☒ Confirm that you have deposited or provided access to graph files (e.g. BED files) for the called peaks.

Data access links *May remain private before publication.*	https://www.ncbi.nlm.nih.gov/geo/query/acc.cgi?acc=GSE268719
Files in database submission	all raw sequencing reads and a table with regions and quantifications
Genome browser session (e.g. UCSC)	none

Methodology

Replicates	2 independent replicates per condition for the vast majority of factors, as detailed in the methods.
Sequencing depth	libraries have sequenced to an average of 16.8 million mapped reads
Antibodies	anti-GATA6 (Cell Signaling technology #5851); anti-OCT4 (abcam ab19857); anti-SOX2 (Active Motif 39823); anti-ESRRB (Perseus Proteomics, H6-705-00); anti-SOX17 (R&D Systems AF1924)
Peak calling parameters	standard MACS2 followed by FDR filtering and replicate reproduction, as described in the methods
Data quality	excellent, assessed by fastqc and reproducibility of published data
Software	none developed for this study (Bowtie2, MACS2, bamsignals, R3.6.3)
